# The distribution of plasmids that carry virulence and resistance genes in *Staphylococcus aureus* is lineage associated

**DOI:** 10.1186/1471-2180-12-104

**Published:** 2012-06-12

**Authors:** Alex J McCarthy, Jodi A Lindsay

**Affiliations:** 1 Centre for Infection, Division of Clinical Sciences, St George’s, University of London, London, UK

## Abstract

**Background:**

*Staphylococcus aureus* is major human and animal pathogen. Plasmids often carry resistance genes and virulence genes that can disseminate through *S. aureus* populations by horizontal gene transfer (HGT) mechanisms. Sequences of *S. aureus* plasmids in the public domain and data from multi-strain microarrays were analysed to investigate (i) the distribution of resistance genes and virulence genes on *S. aureus* plasmids, and (ii) the distribution of plasmids between *S. aureus* lineages.

**Results:**

A total of 21 plasmid *rep* gene families, of which 13 were novel to this study, were characterised using a previously proposed classification system. 243 sequenced plasmids were assigned to 39 plasmid groups that each possessed a unique combination of *rep* genes. We show some resistance genes (including *ermC* and *cat*) and virulence genes (including *entA*, *entG*, *entJ*, *entP*) were associated with specific plasmid groups suggesting there are genetic pressures preventing recombination of these genes into novel plasmid groups. Whole genome microarray analysis revealed that plasmid *rep*, resistance and virulence genes were associated with *S. aureus* lineages, suggesting restriction-modification (RM) barriers to HGT of plasmids between strains exist. Conjugation transfer (*tra*) complex genes were rare.

**Conclusion:**

This study argues that genetic pressures are restraining the spread of resistance and virulence genes amongst *S. aureus* plasmids, and amongst *S. aureus* populations, delaying the emergence of fully virulent and resistant strains.

## Background

Methicillin-resistant *Staphylococcus aureus* (MRSA) are versatile and highly adaptive bacteria that are a major cause of hospital-associated (HA) infections, and are emerging to be a common cause of community-associated (CA) and livestock-associated (LA) infections. Resistance to every antibiotic commonly prescribed is reported, and therefore the treatment and control of MRSA populations is difficult; this is of global concern. Resistance and virulence genes are often carried on mobile genetic elements (MGEs), such as bacteriophage, plasmids and transposons [1,2]. Dissemination of these genes through *S. aureus* populations by horizontal gene transfer (HGT) will lead to strains that are both more resistant and more virulent [1].

Plasmids carry a diverse range of antimicrobial and biocide resistance genes and can carry toxin genes [2-4]. Resistances to antimicrobial agents carried by *S. aureus* plasmids include aminoglycosides, β-lactams and macrolides. Recently, the sequencing of *S. aureus* plasmids originating from different bacterial environments has revealed novel resistance genes, such as the *apmA* and *vgaC* genes encoding resistance to apramycin and streptogramin A, respectively [5,6]. In addition, heavy metal resistance genes are often carried on plasmids [7]. Toxin genes carried on *S. aureus* plasmids include exotoxin B (ETB), a toxin that causes blistering of the skin, and the toxins EntA, EntG, EntJ and EntP [8].

The classification of plasmids has historically been determined by incompatibility groups based on the finding that two plasmids with the same replication (Rep) proteins cannot be stably maintained in the same cell [9,10]. More recently this method has been developed based on the sequence of the *rep* genes [11]. The sequence of a large number of plasmids isolated from *S. aureus* has now been released into the public domain; however there is currently no clear understanding of how virulence genes and resistance genes are linked to *rep* genes and plasmids. Such knowledge is fundamental in understanding the spread of resistance and virulence.

Additional barriers to the spread of plasmids between bacteria are the restriction-modification (R-M) systems. Two systems have been described in *S. aureus*; the type III R-M system protects bacteria against foreign DNA originating from other bacterial species [12], whilst the type I (*SauI*) R-M system protects bacteria against DNA originating from isolates of different *S. aureus* lineages [13]. The type I RM system consists of a restriction subunit (HsdR) and a modification subunit (HsdM) that can cleave and methylate DNA, and a specificity subunit (HsdS) that determines the specificity of the restriction and modification. Each lineage of *S. aureus* encodes unique sequence specificity *hsdS* genes; and this means that DNA originating from different lineages by HGT is detected as foreign DNA and is digested, whilst DNA originating from the same lineage is detected as self DNA and remains undigested. Therefore, exchange of MGEs between lineages is infrequent [13]. Human *S. aureus* can be grouped into 10 major clonal complex (CC) lineages and many minor lineages [14]. Each lineage has a unique but highly conserved combination of genes encoding surface and secreted proteins [15]. However, there is much variation in the carriage of MGEs within a lineage suggesting that HGT is frequent within a *S. aureus* lineage [16,17].

Our specific aims of this study were (i) to extend the *rep* family classification to 243 sequenced *S. aureus* plasmids, (ii) to characterise the distribution of *rep* genes amongst the sequenced plasmids, (iii) to assess the distribution of 45 resistance and virulence genes between plasmids, and (iv) to investigate the distribution of plasmids between 254 *S. aureus* isolates from 20 different lineages using microarray analysis. The overall aim was to better understand the dissemination of plasmids, resistance and virulence genes in *S. aureus* populations. We report 39 unique plasmid groups each with a unique combination of *rep* genes, and demonstrate that resistance and virulence genes are associated with plasmid groups and with lineage. Both of these findings suggest that genetic pressures are restraining the evolution of increasingly resistant and virulent *S. aureus* strains.

## Results

### Characterisation of *rep* families

A total of 21 *rep* families were assigned. 8 families (*rep*_5_*rep*_7_*rep*_10_*rep*_10b_*rep*_13_*rep*_15_*rep*_16_ and *rep*_19_) match those previously characterised by Jensen *et al.*[11]. 13 *rep* families are newly characterised in this study. 6 orphan *rep* sequences were also identified; in plasmids pAVX (repA_N domain), pWBG746 (repA_N domain), pWBG745 (repA_N domain), pKKS825 (rep_1 domain), pRJ6 (rep_3 domain), SAP099B (rep_2 domain).

### Plasmid groups possess unique combinations of *rep* genes

**Figure 1:**
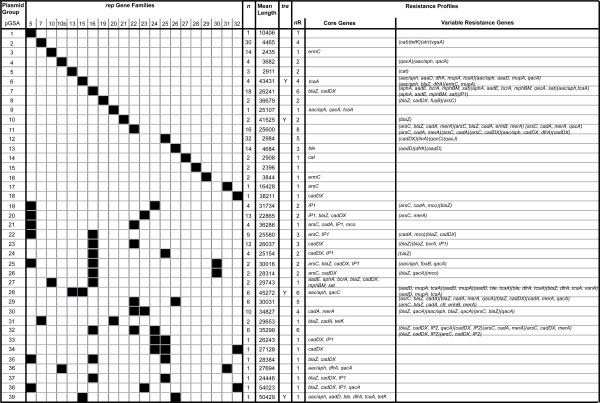
**The distribution of*rep*genes and resistance genes in*S. aureus*plasmids.** Sequenced plasmids may carry a single *rep* gene or a combination of *rep* genes. Each unique *rep* gene combination forms a plasmid group of *S. aureus* (*p*GSA). The number (*n*) and average length (nucleotides) of plasmids in each plasmid group is shown. Plasmid conjugation transfer (*tra*) genes are present in single-*rep* plasmid groups that possess *rep*_15_ and *rep*_21_ genes. The number (*nR*) of resistance gene profiles carried by members of each plasmid group is shown. Core resistance genes are found in all plasmids of a plasmid group, variable resistance genes are found in only some plasmids of the group.

A total of 39 plasmid groups of *Staphylococcus aureus* (p*GSA*) were assigned (Figure 1) based on the combination of *rep* genes each plasmid possessed. Each plasmid group had a unique combination of *rep* gene sequences. 6 of the 243 sequenced plasmids contain orphan *rep* sequences and were not assigned to a plasmid group. 18 plasmid groups carried 1 *rep* sequence, 17 plasmid groups carried 2 *rep* sequences and 4 plasmid groups carried 3 *rep* sequences. The large number of plasmid groups with more than 1 *rep* gene indicates high levels of recombination between *S. aureus* plasmids. We note that in the majority of cases there was no difference in the length of a *rep* gene that appeared on single *rep* plasmids or multi-*rep* plasmids. The number of plasmids belonging to each plasmid group varied considerably (ranging from 1–32). The average length of plasmids belonging to plasmid groups varied (Figure 1). Nine plasmid groups have small genomes (<5Kb) and carried few genes. 28 plasmid groups have large genomes (>15Kb) and carried a diverse range of genes. 21 of these 28 large plasmid groups possessed more than 1 *rep* gene sequence. Many of these large plasmids carried *rep* genes found in small plasmids indicating recombination and integration of smaller plasmids. 13/243 plasmids carried plasmid conjugation transfer (*tra*) A-M genes. All plasmids from groups *pGSA*_6_, *pGSA*_28_ and *pGSA*_39_ possessed *tra*A-M genes, whilst plasmids from group *pGSA*_10_ possess homologs of *traE*, *traG* and *traI*. Conjugation ability is therefore tightly linked with the replication machinery and *rep* sequences of *rep*_15_ and *rep*_21_, respectively.

### Resistance genes and virulence genes are associated with plasmid groups

The distribution of antimicrobial resistance, biocide resistance and heavy metal resistance genes found on plasmids was investigated (Additional file 1). The same resistance gene profile was found amongst all members of 16 plasmid groups (Figure 1). For example, small plasmids belonging to *pGSA*_3_ all carried the *ermC* gene, and differed only by SNPs and insertions and deletions suggesting they are clonal (Figure 1 and Additional file 1). However, in 5 other small plasmid groups completely different resistance gene profiles existed. For example, the 30 plasmids belonging to the *pGSA*_2_ plasmids carried either *cat*, *tetK*, *str* or *vgaA*. In contrast, larger plasmids carried more resistance genes, and 23 plasmid groups had more than one resistance gene profile. The majority of variation within these plasmid groups was due to the addition of resistance genes to a set of core conserved resistance genes or due to different combinations of the same resistances. For example, *pGSA*_7_ plasmids carried *blaZ* and *cadDX* with or without *aac/aph, aadE, aphA*, *bcrA*, *IP1*, *mphBM*, *qacA*, *sat* and *tcaA* (Figure 1 and Additional file 1).

Toxin genes were rare amongst the sequence plasmids. ETB was only found in pETB. The genes *entA*, *entG* and *entJ* were tightly associated with *pGSA*_23_ (present in 10/12 plasmids). These genes were also present in a single member of the *pGSA*_29_ group suggesting that these genes can move to other plasmids. *entP* was associated with *pGSA*_32_ (present in 4/6 plasmids). Interestingly, these toxin genes were most frequently found on plasmids carrying more than 1 *rep* gene.

Some resistance genes had strong associations with particular *rep* genes and plasmid groups. The tetracycline resistance gene *tetK* was found in *pGSA*_2_ plasmids indicating that the gene is tightly linked with the *rep*_7_ gene (Figure 1). The chloramphenicol resistance gene *cat* was found only in *pGSA*_2_, *pGSA*_5_ and *pGSA*_14_ plasmids. Other resistance genes were not associated with particular *rep* genes or plasmid groups; *arsC*, *blaZ*, *cadDX*, *qacA*.

### Microarray analysis reveals that *rep*, resistance and virulence genes are associated with *S. aureus* lineage

**Figure 2:**
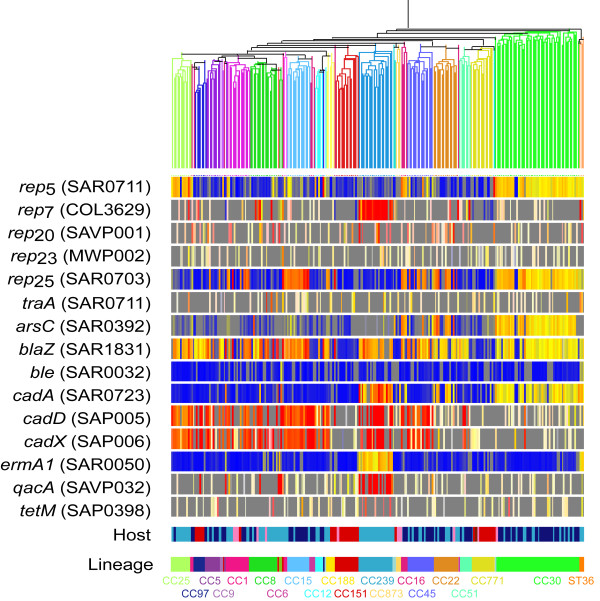
**Distribution of plasmids in 254** ***S. aureus*(198 human isolates and 55 animal isolates) using microarray.** Presence or absence of each gene (listed on left) in each isolate is depicted by colour. The colour is an indicator of test signal over reference signal ratio. Thus, (i) yellow indicates presence of the gene in both test strain and reference strain, (ii) red indicates presence of the gene in the test strain but not in the reference strain, (iii) blue indicates absence in the test strain but not the reference strain, and (iv) grey indicates absence in both the test and reference strains. Genes with white signals are very low intensity and regarded as negative for both strains. The colour intensity is an indicator of signal intensity, and this can differ because (i) the homology of the probe, which can be hundreds of base pairs long, and DNA may vary, and (ii) copy numbers may vary. Isolates (represented vertically) are clustered into lineages [14]. For each isolate, its mammalian host of origin and its lineage (clonal complex) are shown at the bottom of the figure. Human isolates are coloured light blue (invasive) and dark blue (carriage). Animal isolates are coloured red (cow), pink (horse), maroon (sheep and goat) and white (camel). The figure shows that *rep* genes and resistance genes are distributed in a lineage dependent manner.

Microarray analysis showed that there was a differential distribution of 4/5 *rep* genes represented on the microarray (*rep*_5_, *rep*_7_, *rep*_20_ and *rep*_25_) (Figure 2). *rep*_5_ genes were found in isolates belonging to *S. aureus* lineages CC15, CC25, CC30 and CC45 but were rare in other major lineages. *rep*_7_ gene was commonly found in CC239 *S. aureus*, but was rare in other major lineages. *rep*_20_ was found commonly in CC22 isolates. *rep*_25_ was found *S. aureus* isolates belonging to lineages CC1, CC15, CC22, CC30 and CC45, but was rare in other lineages. *rep*_23_ were rare in all the *S. aureus* isolates included in our analysis. This analysis demonstrates an association of *rep* genes with *S. aureus* lineages. This is likely to be driven by both clonal expansion and by more frequent HGT within lineages than between lineages.

We also assessed the distribution of other plasmid genes between *S. aureus* lineages. The presence of plasmid conjugation transfer (*tra*)A-M genes was rare amongst the *S. aureus* isolates in our collection and was not associated with lineage (Figure 2). Interestingly, antimicrobial resistance genes and heavy metal resistance genes were associated to lineage. *arsC* was common in MRSA CC22 and CC30 isolates, but rare amongst other lineages. *blaZ* was common in all human lineages of *S. aureus* but was rare in animal lineages of *S. aureus*. *cadA* presence was associated with MRSA CC22, CC30 and CC239 lineages, whilst *cadDX* was widely distributed and associated with 9 different lineages. *ermA* presence was associated with CC8 and CC239 lineages. *qacA* was associated with CC239 lineage. 2 of 9 (*ble* and *tetM*) resistance genes represented on the microarray are rare in the isolates we have analysed and were not distributed in a lineage dependent manner. We note that some of these genes may be carried on other elements or on integrated plasmids and this cannot be determined by microarray alone, for example *tetM* can also be carried on transposons such as Tn*5801*.

## Discussion

In this study we extended a previously proposed plasmid classification system to characterise *rep* genes from 243 plasmids that appear in the public domain [11]. We characterised 21 *rep* families, of which 13 are newly described in this study. Whilst performing this analysis we noted that many plasmids carried more than one *rep* gene, we therefore assigned plasmids into groups based on the combination of *rep* genes carried. A total of 39 plasmid groups were assigned, and interestingly 20/39 groups of sequenced plasmid carry more than one *rep* gene sequence. This indicates that recombination between *S. aureus* plasmids has occurred frequently. Recombination between *S. aureus* plasmids has been described, but the mechanisms and the frequency of such recombination events is not clearly understood [18]. Recombination should be a mechanism that transfers virulence and resistance genes into new plasmid groups.

The highly mosaic structure of plasmids seen suggests frequent recombination, but if this was completely random then resistance and virulence genes would not be associated to particular plasmid groups. Surprisingly, this was not the case. We found that some resistance and virulence genes were associated with plasmid groups; for example all *pGSA*_3_ carried the *ermC* gene. This suggests there are tight associations between particular *rep* and resistance gene combinations. Resistance and virulence genes that had wider plasmid distributions were typically located on transposable elements that can “hop” between plasmids. This included *blaZ* located on Tn*552* and *cadDX* on insertion sequence (IS) elements [19,20].

We also found evidence of movement of genes tightly linked to specific plasmids; (i) the virulence genes *entA*, *entG* and *entJ* are tightly linked with *pGSA*_23_, but were also found in a single plasmid that belongs to *pGSA*_29_, and (ii) the bacitracin resistance gene *bcrA* that is tightly linked to the *pGSA*_7_ plasmids, was also found in 1/12 *pGSA*_23_ plasmids. This argues that recombination can disseminate resistance and virulence genes into new plasmids, though this is rare.

Why is plasmid recombination not completely random? Recombination is likely to generate non-functional plasmids, or novel plasmids that cannot out-compete their parental plasmids. Because of the RM system it is possible that some plasmids do not come into contact because they are restricted to a small number of lineages. Some plasmids will be selected for because they provide a benefit to their hosts in specific environments. In addition, plasmids may be incompatible and this means that certain plasmids may not survive well in the same cell.

Indeed, this study also showed that the distribution of plasmids in *S. aureus* is lineage associated. This could limit the opportunities for plasmids in different lineages to recombine. There are two possible explanations for lineage associations of plasmids. Firstly, plasmids are distributed by clonal expansion and passed to daughter cells during replication. We found evidence that this occurs frequently, such as the CC239 isolates included in our analysis which represent a single dominant clone of invasive MRSA from a hospital in London, U.K. [21]. All isolates carried the same *rep* genes; this is evidence that clonal expansion can be a cause of plasmid distributions being lineage associated. Our conclusions are supported by the recent finding that USA300 (CC8) isolates carried highly conserved plasmids [22]. The second explanation is that plasmids are transferred between isolates frequently, but are blocked by efficient RM barriers, reducing transfer between isolates of different lineages. We found evidence that this occurs in *S. aureus* populations. Many plasmids were lineage associated but only found in some isolates, including those from different times and locations, indicating loss of plasmids as well as transfer.

The plasmids and resistances carried by our *S. aureus* isolates are reflective of the selective exposures existing in U.K. environments. Isolates originating from different countries may belong to different lineages and come into contact with the different exposures and carry different plasmids and resistances, or carry them at different frequencies [23]. Antibiotic usage and host specific plasmids are therefore also likely to have roles in controlling plasmid dissemination. The sequenced *S. aureus* plasmids may not be representative of all plasmid diversity, as they originate from a small number of lineages from only a few countries.

It is generally accepted that plasmids that contain the same origin of replication are incompatible and cannot survive within the same cell [9,10]. This study has identified a diverse range of *rep* genes and *rep* gene combinations. Biological tests are required to determine the incompatibility of plasmid groups, and to draw conclusions on the importance of this phenomenon in limiting plasmid recombination.

MGEs in other bacterial species may be additional sources of novel resistance and virulence genes that can move into *S. aureus* populations. Importantly, the *vanA* gene in vancomycin-resistant *S. aureus* (VRSA) isolates is carried on a transposon Tn*1546* which is commonly found in vancomycin-resistant enterococci [24,25]. In some VRSA isolates the entire Enterococcal plasmid has been maintained, whilst in others Tn*1546* has moved onto a Staphylococcal plasmid. Both genetic events suggest that enterococcal plasmid have successfully transferred into *S. aureus* bacteria. Future studies are required that assess the mosaicism of Staphylococcal and Enterococcal plasmids in order to understand the frequency of recombination and gene exchange between such bacterial species.

HGT mechanisms spread resistance and virulence genes between bacteria and populations. In *S. aureus*, two major HGT mechanisms have been described for plasmid movement (i) plasmid conjugation via the conjugation transfer (*tra*) complex, and (ii) bateriophage generalized transduction. In addition, it is possible that smaller plasmids can hitchhike larger plasmids that carry the *tra* complex and be transferred from donor to recipient bacteria [26]. We found that the *tra* genes were rare amongst the sequenced plasmids (13/243) and were rare amongst our collection of 254 *S. aureus* isolates. Bacteriophage generalized transduction can transfer DNA fragments of less than 45Kb. We found that 96.7 % of plasmids could theoretically be transferred by generalized transduction as they have genomes that are <45Kb in length. 6 of the 8 plasmids >45Kb in length carry the *tra* genes. Collectively, this data suggests that conjugative plasmids and plasmid conjugation are infrequent, and that bacteriophage transduction is likely to be the most frequent transfer mechanism of plasmids, particularly non-conjugative plasmids.

## Conclusion

Plasmids are a principal driver of the spread of virulence and resistance genes in *S. aureus* populations via HGT, which is blocked by lineage specific R-M systems. This study has demonstrated that resistance and virulence genes are associated with plasmid groups, and that plasmids are associated with *S. aureus* lineage. This is evidence that genetic pressures and RM barriers are limiting the evolution of more resistant and more virulent *S. aureus* strains.

## Methods

### Plasmid sequences

A total of 243 sequenced *S. aureus* plasmids obtained from GenBank were included in analysis. 47 of these sequences are isolated from contigs of whole genome sequencing projects. GenBank accession numbers for all plasmid sequences are shown in Additional file 1. The lineage origin of plasmids is unknown for the majority of these plasmids, and therefore distributions of sequenced plasmid amongst lineages could not be investigated.

### *rep* gene assignment

*rep* genes were identified by the presence of previously characterised protein replication domains (rep_1, rep_2, rep_3, repA_N, repL and rep_trans) using the protein-protein BLAST search (http://www.ncbi.nlm.nih.gov/blast) [4]. Because *rep* genes can appear in truncated forms, those that encode proteins of less than 90 amino acids in length were not included in analysis. A *rep* family was assigned if two distinct *rep* gene sequences from two different plasmids shared at least 80 % amino acid identity across the whole gene, as previously performed by Jensen *et al.*[11]. All *rep* families were named *rep*_*X*_ with the *X* indicating the designated number of the family, and match those previously described by Jensen *et al.* 2009. *rep* genes that were identified in only one *S. aureus* plasmids were termed *rep* orphans.

### Assignment of plasmid groups

A plasmid group was assigned to each unique combination of *rep* genes found in a single sequenced plasmid. All plasmid groups were named *pGSA*_*X*_ (for plasmid group of *Staphylococcus aureus*) with the *X* indicating the designated number of the family. All members of the same plasmid group share the same *rep* gene or genes. Plasmid groups exist that possess a single *rep* gene. Other plasmid groups possess more than one *rep* gene.

### Distribution of resistance, virulence and transfer genes in *S. aureus* plasmids

The distribution of genes carried on plasmids that have characterised or hypothesised roles in antimicrobial resistance (*n* = 29), biocide resistance (*n* = 3), heavy metal resistance (*n* = 5), transfer (*n* = 17), toxicity (*n* = 5) or adherence (*n* = 2) in sequenced plasmids was assessed by BLAST analysis of a representative gene sequence; a gene was present in a plasmid if there was 95 % amino acid sequence identity. The genes and their characterised roles are shown in Table 1.

**Table 1:** **Genes carried on plasmids involved in*S. aureus*survival and adaptation**

**Gene Class**	**Gene**	**Accession Number/ Locus Tag**	Function
Antimicrobial resistance, biocide resistance and heavy metal resistance	*aacA/aphD*	VRA0030	Gentamicin & Kanamycin Resistance
	*aadD*	PGO1_p21	Neomycin & Kanamycin Resistance
	*aadE*	SAP049A_002	Aminoglycoside Resistance
	*aphA*	SAP049A_001	Neomycin & Kanamycin Resistance
	*arsC*	SAP013A_020	Arsenic Resistance
	*bcrA*	SAP049A_007	Resistance to Bacitracins
	*blaZ*	pBORa53p07	Penicillin Resistance
	*ble*	PGO1_p20	Bleomycin Resistance
	*cadA*	SATW20_p1220	Cadmium Resistance
	*cadDX*	pKH18_01 _02	Cadmium Resistance
	*cat*	pTZ4_p2	Chloramphenicol Resistance
	*cfr*	EF450709	Chloramphenicol, Lincosamides & Linezolid Resistance
	*dfrA*	PGO1_p48	Trimethoprim Resistance
	*dfrK*	FN377602	Trimethoprim Resistance
	*ermB*	SAP013A_023	MLS Group Resistance
	*ermC*	pKH19_p2	MLS Group Resistance
	*fosB*	pTZ2162_25	Fosomycin Resistance
	*fusB*	pUB101_p23	Fusidic Acid Resistance
	*IP1*	pBORa53p09	Immunity Protein
	*IP2*	SAP099A_005	Immunity Protein
	*linA*	pKH21_p2	Linezolid Resistance
	*mco*	SAP019A_028	Copper Resistance
	*merA*	SAP026A_033	Mercury Resistance
	*mphBM*	SAP052A_035	Macrolide Resistance
	*mupA*	SAP082A_042	Mupirocin Resistance
	*qacA*	SAP066A_020	Biocide Resistance
	*qacC*	VRA0026	Biocide Resistance
	*qacJ*	pNVH01_p2	Biocide Resistance
	*sat*	SAP049A_002	Streptothricin Resistance
	*str*	pS194_p1	Streptomycin Resistance
	*tcaA*	SAP082A_032	Teichoplanin Resistance
	*tetK*	pKH17_02	Tetracycline Resistance
	*tetL*	FN377602	Tetracycline Resistance
	*tetM*	SAPIG0957	Tetracycline & Minocycline Resistance
	*vanB*	VRA0040	Vancomycin Resistance
	*vatA*	M36022	Streptogramin Resistance
	*vgaA*	pVGA_p2	Streptogramin Resistance
	*vgaB*	U82085	Streptogramin Resistance
Transfer	*traA*	SAP082A_013	Plasmid conjugation
	*traB*	SAP082A_012	Plasmid conjugation
	*traC*	SAP082A_011	Plasmid conjugation
	*traD*	SAP082A_010	Plasmid conjugation
	*traE*	SAP082A_009	Plasmid conjugation
	*traF*	SAP082A_008	Plasmid conjugation
	*traG*	SAP082A_007	Plasmid conjugation
	*traH*	SAP082A_006	Plasmid conjugation
	*traI*	SAP082A_005	Plasmid conjugation
	*traJ*	SAP082A_004	Plasmid conjugation
	*traK*	SAP082A_003	Plasmid conjugation
	*traL*	SAP082A_002	Plasmid conjugation
	*traM*	SAP082A_001	Plasmid conjugation
	*type III R-M*	SAP039A_002	Prevents Survival of Foreign DNA in Host Bacterium
	*mob-I*	AF447813	Mobilisation L gene
	*cas3*	SAP039A_001	Helicase of the CRISPR region
	*abiK*	SAP058A_004	Prevents Bacteriophage Replication
	*C55*	pETB_p42	Lantibiotic System that Kills other Bacteria
Toxins	*ETB*	pETB_p01	Toxin
	*entA*	SAP048A_010	Toxin
	*entG*	SAP048A_007	Toxin
	*entJ*	SAP048A_008	Toxin
	*entP*	SAP099A_058	Toxin
Adherence	*sdrE*	SAP041A_028	Adherence to Host Cells
	*Anti-adhesin*	SAP057A_026	Prevents Adherence

*MLS*, Macrolide & Streptogramins. *CRISPR*, clustered regularly interspaced short palindromic repeats.

### Distribution of plasmid genes in *S. aureus* lineages

In order to investigate the distribution of plasmid genes between *S. aureus* from diverse lineages we further analysed previous microarray data we generated from 254 human and animal *S. aureus* isolates of U.K. origin. The 198 human carriage and invasive isolates have been previously described and represent the major dominant lineages of *S. aureus* from hospitals and the community [14,21,27]. The 55 animal isolates have previously been described and originate from cows (*n* = 37), horses (*n* = 13), sheep (*n* = 2), goats (*n* = 2) and a camel (*n* = 1) [28]. The array design is available in BμG@Sbase (accession number: A-BUGS-17; httpbugs.sgul.ac.uk/A-BUGS-17) and also ArrayExpress [28] and represents all the predicted ORFs from the first seven whole-genome *S. aureus* sequencing projects publically released, including five *rep* genes. Experiments were performed as previously reported [28]. The data used here is deposited in BμG@Sbase (accession number: E-BUGS-62 and E-BUGS-34) and also ArrayExpress (accession number: E-BUGS-62 and E-BUGS-34).

Microarrays are an accurate, but not 100 % accurate, way of determining presence and absence of individual genes in individual isolates using a single experiment. A full discussion of this accuracy is provided in Witney *et al*. [28]. Microarray heatmaps are an appropriate way to show microarray data as they accurately display the ratio of test signal and reference signal for each individual isolate. By analyzing multiple isolates from the same lineage it is possible to determine if genes are associated with individual lineages [14,27].

## Authors’ contributions

AJM participated in study design, performed all analysis and drafted the manuscript. JAL participated in the study design and manuscript revisions. All authors read and approved the final manuscript.

## Authors’ information

AJM is a Post-Doctoral Research Fellow at St George’s, University of London interested in pathogen evolution and host-pathogen interactions of bacteria and viruses. JAL is a Reader in Microbiol Pathogenesis interested in *S. aureus*.

## Supplementary Material

Additional file 1 **Distribution of rep, resistance, transfer, toxin and adherence genes in sequenced plasmids.** Description: Presence of *rep* genes in all sequenced plasmids is shown by a black box, whilst a white box indicates absence. Plasmids are classified into plasmid groups by the combination of rep sequences that they carry. The presence of resistance, transfer, toxin and adherence genes is shown by “Y”. Plasmids that originate from a whole genome sequencing contig are marked by *. Click here for file
